# 

**Published:** 1974-07

**Authors:** 


The
Bristol Medico-Chirurgical Journal
(Incorporating the Medical Journal of the South-West)
Established 1883

Vol. 89 (iii) JULY 1974 No. 331

### BRISTOL MEDICO-CHIRURGICAL JOURNAL

Editor: N. J. Brown, M. B., F.R.C.P., F.R.C.Path.

Published Quarterly

Annual Subscription £2 Post Free

### BRISTOL MEDICO-CHIRURGICAL SOCIETY

**President 1973-74: Dr. R. F. Barbour**

**President-elect: Dr. J. Apley**

**Honorary Secretary: Dr. I. S. Bailey, 7 Percival Road, Bristol 8.**

**Honorary Treasurer: Dr. Frank Ross, Bristol Royal Infirmary, Bristol 2.**

The Society was founded in 1874. In 1883 publication of the Journal began and has continued quarterly ever since then. During the years 1949 to 1962 it appeared under the title of "The Medical Journal of the South West".

### THE BRISTOL MEDICO-CHIRURGICAL JOURNAL

**Editorial Committee**

| | |
|---|---|
| Editor | Dr. N. J. Brown, Southmead Hospital, Bristol. |
| Assistant Editor | Dr. D. S. Reeves. |
| Members | Dr. Rhys Davies.<br>Dr. M. B. Lennard.<br>Professor R. C. Wofinden.<br>Dr. D. W. Wright.<br>The Hon. Treasurer and Hon. Secretary of the Society. |
| Business Manager | Dr. P. J. F. Baskett, Frenchay Hospital, Bristol. |
| Secretary | Mr. B. P. Jones, The Library, Medical School, University Walk, Bristol BS8 1TD. |

### NOTICE TO CONTRIBUTORS

The Journal is especially concerned to provide a place for the recording of medical thought, interests, and practice in and around Bristol. It therefore welcomes articles that, as well as being of immediate interest to members, will document the local and contemporary medical scene for our successors.

Original articles are invited provided that they have not been published elsewhere. References in the text should be by author's name, with year of publication in parenthesis; they should be listed at the end in alphabetical order, the name of each journal or book being given in full—not abbreviated—and the title of the article added. Illustrations should be supplied ready for reproduction. Line drawings should be in Indian ink on firm white paper or board. The author will be informed if it is found necessary to ask him to pay for the making of blocks.

The following contributions will be especially welcome; notes of forthcoming events, meetings held, degrees conferred, staff appointments, honours and public appointments and other local news.

### Bristol Medico-Chirurgical Society

*PRESIDENTS 1874-1973*

| | | | | | |
|---|---|---|---|---|---|
| 1874 | Dr. F. Brittan | 1897 | Dr. J. E. Shaw | 1920 | Dr. L. E. V. Every-Clayton |
| 1875 | Mr. R. W. Coe | 1898 | Dr. R. Roxburgh | 1921 | Mr. C. H. Walker |
| 1876 | Dr. S. Martyn | 1899 | Mr. W. H. Harsant | 1922 | Dr. J. A. Nixon |
| 1877 | Mr. A. Prichard | 1900 | Dr. D. S. Davies | 1923 | Dr. J. L. Firth |
| 1878 | Dr. H. E. Fripp | 1901 | Dr. B. J. Baron | 1924 | Dr. J. O. Symes |
| 1879 | Mr. W. M. Clarke | 1902 | Mr. G. Munro Smith | 1925 | Mr. T. Carwardine |
| 1880 | Dr. J. W. Swayne | 1903 | Mr. J. Paul Bush | 1926 | Mr. A. L. Fleming |
| 1881 | Dr. E. L. Fox | 1904 | Dr. R. Eager | 1927 | Dr. W. K. Wills |
| 1882 | Dr. J. G. Davey | 1905 | Mr. J. Dacre | 1928 | Dr. H. L. Ormerod |
| 1883 | Dr. W. J. Fyffe | 1906 | Mr. J. Taylor | 1929 | Mr. C. F. Walters |
| 1884 | Dr. G. F. Burder | 1907 | Dr. H. Waldo | 1930 | Dr. D. C. Rayner |
| 1885 | Dr. W. H. Spencer | 1908 | Dr. J. M. Clarke | 1931 | Dr. J. R. Charles |
| 1886 | Mr. F. P. Lansdown | 1909 | Dr. J. Swain | 1932 | Mr. E. W. Hey Groves |
| 1887 | Mr. L. M. Griffiths | 1910 | Dr. B. M. H. Rogers | 1933 | Dr. H. E. Harris |
| 1888 | Dr. R. S. Smith | 1911 | Mr. C. A. Morton | 1934 | Professor A. Rendle Short |
| 1889 | Mr. N. G. Dobson | 1912 | Dr. W. C. Swayne | 1935 | Dr. A. E. Iles |
| 1890 | Mr. S. H. Swayne | 1913 | Dr. P. Watson-Williams | 1936 | Dr. R. C. Clarke |
| 1891 | Mr. F. Richardson Cross | 1914 | Dr. W. H. C. Newnham | 1937 | Mr. C. A. Moore |
| 1892 | Dr. E. Markham Skerritt | 1915 | Dr. G. Parkes | 1938 | Dr. L. Moore |
| 1893 | Mr. J. Greig Smith | 1916 | Major F. H. Edgeworth | 1942 | Dr. C. Corfield |
| 1894 | Dr. A. J. Harrison | 1917 | Mr. F. Lace | 1945 | Dr. H. H. Carleton |
| 1895 | Mr. A. W. Prichard | 1918 | Major R. G. P. Lansdown | | |
| 1896 | Dr. A. E. Aust Lawrence | 1919 | Professor I. Walker Hall | | |

| | | *TITLE OF PRESIDENTIAL ADDRESS* |
|---|---|---|
| 1946 | Mr. H. Chitty | Back to Work |
| 1947 | Dr. T. Milling | The Life of Edward Jenner |
| 1948 | Professor H. J. Drew-Smythe | Obstetrics, gynaecology: old and new |
| 1949 | Dr. H. J. Orr-Ewing | Coronary Thrombosis |
| 1950 | Mr. W. A. Jackman | Thirty Years of Surgery |
| 1951 | Dr. Victoria Tryon | Women Physicians |
| 1952 | Dr. G. E. F. Sutton | Damned Drugs |
| 1953 | Mr. R. Ramsey Garden | Blindness |
| 1954 | Dr. A. G. Heron | That backbone—general practice |
| 1955 | Dr. P. Phillips | Medical Worthies of the South-West |
| 1956 | Dr. E. G. Bradbeer | The Progress of Anaesthesia |
| 1957 | Professor C. Bruce Perry | Medical Education |
| 1958 | Mr. H. L. Shepherd | Midwifery in Bristol |
| 1959 | Dr. F. W. Terrell Hughes | The Lure and Beauty of High Places |
| 1960 | Professor R. J. Brocklehurst | General Medical Council |
| 1961 | Mr. J. Angell James | The Use of Otorhinolaryngology |
| 1962 | Dr. A. M. Maclachlan | The Unorthodox in Medicine and Religion |
| 1963 | Mr. R. V. Cooke | Bristol Fashion |
| 1964 | Dr. G. L. Feneley | Anaesthesia |
| 1965 | Mr. G. M. Fitzgibbon | Pictorial Art in Medicine |
| 1966 | Dr. S. F. Marwood | Louis Napoleon |
| 1967 | Professor T. F. Hewer | Flowers |
| 1968 | Mr. A. L. Eyre-Brook | History of B.R.I. |
| 1969 | Dr. F. Lewis | Leukaemia. Cytogenetics |
| 1970 | Dr. Beryl Corner | Beethoven |
| 1971 | Dr. C. C. Morgans | St. Peter |
| 1972 | Dr. J. Macrae | Jenner |
| 1973 | Dr. R. F. Barbour | The History of Psychiatry in Bristol |

### HONORARY SECRETARIES

| | | | | | |
|---|---|---|---|---|---|
| 1874 | Dr. R. Shingleton Smith | 1916 | Mr. A. L. Fleming | 1946 | Mr. A. L. Eyre-Brook |
| 1888 | Mr. G. Munro Smith | 1918 | Mr. A. J. Wright | 1952 | Dr. B. McConnell |
| 1894 | Mr. J. P. Bush | 1920 | Mr. S. V. Stock | 1960 | Dr. A. T. M. Roberts |
| 1903 | Mr. H. F. Mole | 1926 | Mr. W. A. Jackman | 1968 | Dr. I. S. Bailey |
| 1907 | Dr. J. A. Nixon | 1931 | Mr. H. L. Shepherd | | |
| 1912 | Mr. A. J. Wright | 1935 | Mr. R. V. Cooke | | |

### HONORARY TREASURERS

*(appointed following a change in the rules in 1926)*

| | | | |
|---|---|---|---|
| 1926 | Dr. A. E. Iles | 1953 | Dr. J. Macrae |
| 1945 | Dr. G. E. F. Sutton | 1969 | Dr. F. G. M. Ross |

### The Centenary Dinner

### BRISTOL MEDICO-CHIRURGICAL SOCIETY

The Sixth Meeting of the session on 13th March, 1974, took the form of a Symposium on 'Population Control' presented by Professor H. G. Dixon, Miss Gillian M. Turner, Mr. G. E. Smart and Dr. R. J. Chandler. Dr. D. H. Johnson showed pathological specimens and Dr. J. B. Penry demonstrated skiagrams.

---

The Guest Speaker at the meeting on 10th April was Sir Martin Roth, President of the Royal College of Psychiatrists who addressed the Society on 'Medicine and Sexual Deviation'. Dr. J. Briggs and Dr. J. L. G. Thomson showed pathological specimens and skiagrams respectively.

---

The Centenary Dinner, reported elsewhere in this number of the Journal, was held on 26th April.

The Final Meeting of the session on 8th May was an open meeting which many non-medical guests attended. Metropolitan Anthony of Sourozh spoke on 'Human Values in Medicine'.

---

At all the above meetings Mr. B. P. Jones, Medical Librarian, provided an appropriate display of medical books.

### UNIVERSITY OF BRISTOL
(Faculty of Medicine)

Ch.M.—A. J. Webb.
M.D. —C. Rosse
Ph.D. —Patricia Brittain
Trudie Anne Gentle

